# Rumination-focused cognitive behaviour therapy vs. cognitive behaviour therapy for depression: study protocol for a randomised controlled superiority trial

**DOI:** 10.1186/s13063-015-0875-y

**Published:** 2015-08-11

**Authors:** Morten Hvenegaard, Ed R. Watkins, Stig Poulsen, Nicole K. Rosenberg, Matthias Gondan, Ben Grafton, Stephen F. Austin, Henriette Howard, Stine B. Moeller

**Affiliations:** Department of Psychology, University of Copenhagen, Øster Farimagsgade 2A, 1353 Copenhagen K, Denmark; School of Psychology, University of Exeter, Sir Henry Wellcome Building for Mood Disorders Research, Streatham Campus, Perry Road, Exeter, EX4 4QG UK; Psychiatric Outpatient Clinic of Copenhagen, Psychiatric Hospital of The Capital Region, Nannasgade 28, 2200 Copenhagen N, Denmark; Centre for the Advancement of Research in Emotion, School of Psychology, University of Western Australia, 35 Stirling Highway, Crawley, WA 6009 Australia; Psychiatric Research Unit, Mental Health Centre North Zealand, Copenhagen University, Dyrehavehavevej 48, 3400 Hillerød, Denmark; Mental Health Centre for Child and Adolescent Psychiatry, Bispebjerg Bakke 30, 2400 Copenhagen, NV Denmark

**Keywords:** Rumination-focused cognitive behavioural therapy, Depression, Rumination, Worry, Relapse prevention, Attentional bias

## Abstract

**Background:**

Cognitive behavioural therapy is an effective treatment for depression. However, one third of the patients do not respond satisfactorily, and relapse rates of around 30 % within the first post-treatment year were reported in a recent meta-analysis. In total, 30–50 % of remitted patients present with residual symptoms by the end of treatment. A common residual symptom is rumination, a process of recurrent negative thinking and dwelling on negative affect. Rumination has been demonstrated as a major factor in vulnerability to depression, predicting the onset, severity, and duration of future depression. Rumination-focused cognitive behavioural therapy is a psychotherapeutic treatment targeting rumination. Because rumination plays a major role in the initiation and maintenance of depression, targeting rumination with rumination-focused cognitive behavioural therapy may be more effective in treating depression and reducing relapse than standard cognitive behavioural therapy.

**Method/design:**

This study is a two-arm pragmatic randomised controlled superiority trial comparing the effectiveness of group-based rumination-focused cognitive behaviour therapy with the effectiveness of group-based cognitive behavioural therapy for treatment of depression. One hundred twenty-eight patients with depression will be recruited from and given treatment in an outpatient service at a psychiatric hospital in Denmark. Our primary outcome will be severity of depressive symptoms (Hamilton Rating Scale for Depression) at completion of treatment. Secondary outcomes will be level of rumination, worry, anxiety, quality of life, behavioural activation, experimental measures of cognitive flexibility, and emotional attentional bias. A 6-month follow-up is planned and will include the primary outcome measure and assessment of relapse.

**Discussion:**

The clinical outcome of this trial may guide clinicians to decide on the merits of including rumination-focused cognitive behavioural therapy in the treatment of depression in outpatient services.

**Trial registration:**

ClinicalTrials.gov Identifier: NCT02278224, registered 28 Oct. 2014.

## Background

Cognitive behavioural therapy (CBT) is a recommended treatment for unipolar depression, and a large number of studies provide supportive evidence for its efficacy in both an individual as well as a group format [1–3]. CBT has been shown to be effective in the short term and to have enduring effects in reducing the risk of returning symptoms after treatment has ended. However, approximately one third of the patients do not respond satisfactorily to the treatment [4, 5], and post-treatment residual symptoms are a common problem; as many as 30–50 % of remitted patients experience residual symptoms at the end of treatment [6]. Residual symptoms of depression are associated with ongoing psychosocial and functional disability and have been demonstrated to predict poorer long-term outcome of major depression, including increased rates of relapse [7–9]. A recent meta-analysis reports relapse rates of around 29 % within 1 year and 56 % within 2 years [10].

Recent studies have evaluated the potential benefit of continuation-phase CBT in reducing residual symptoms as well as in preventing subsequent depressive relapse or recurrence. In general, continuation-phase cognitive therapies have proven to be beneficial in reducing rates of relapse and recurrence [10]. Yet, in most of these studies, only a modest reduction in residual symptoms was observable [11, 12], indicating the possibility of further improvement in efficacy by targeting key residual symptoms. With the aim of improving efficacy, an increased focus has been directed toward a common residual symptom, namely depressive rumination [13, 14]. Rumination is a passive process of recurrent negative thinking and dwelling on negative affect, causes, and symptoms [15] and has been shown to be a major factor in vulnerability to depression as well as predicting the onset, severity, and duration of future depressive episodes [16, 17]. Consequently, if involved in the pathogenesis of depression, rumination may constitute a relevant target for psychological intervention. In support of this, recent psychotherapeutic interventions designed to target depressive rumination show promising results [18–20]. The strongest evidence thus far exists for the rumination-focused cognitive behavioural therapy (RFCBT) developed by Watkins and colleagues [18]. RFCBT is based on a conceptualization of repetitive thinking that differentiates between a functional and a dysfunctional style of thinking. The helpful style is characterized by being concrete and specific, whereas the unhelpful style is abstract and evaluative and does not lead to problem solving (rumination) [21]. In RFCBT, rumination is formulated as a habitual behaviour controlled by the laws of behavioural psychology [22] and maintained by negative reinforcement. Rumination can act as a form of avoidance by thinking about difficulties rather than confronting them directly in the real world and thereby avoiding the risk of failure and negative outcomes. Hence, rumination can become reinforced by escape and avoidance. Grounded in behavioural psychology and research, RFCBT focuses on functional analyses of the target behaviour, rumination, and combines strategies from behavioural activation with novel strategies to foster concrete, process-focused, and specific thinking. RFCBT differs from standard CBT by focusing on modifying the process of thinking, whereas CBT focuses on modifying the content of the thoughts and content of dysfunctional schemas [14]. With only a handful of trials conducted, the evidence for RFCBT is still sparse, although it is promising. For example, in a recent clinical trial with patients with medication-refractory residual depression, 42 patients were randomly allocated (1:1) to treatment-as-usual (TAU) consisting of continuation anti-depressants and outpatient clinical management or to TAU plus up to 12 sessions of individual RFCBT. RFCBT was found to be superior to TAU; 62 % of patients in the RFCBT treatment condition achieved remission, compared with 21 % in the TAU [18]. However, to date, no study has made a direct comparison of RFCBT with standard CBT. The aim of the present study is to compare group-based rumination-focused cognitive behaviour therapy (g-RFCBT) with group-based standard cognitive behavioural therapy (g-CBT) for depression on the effectiveness of treatment and the reduction of relapse rates at 6-month follow-up.

## Methods/design

This study is a two-arm pragmatic randomised controlled trial comparing the effectiveness of g-RFCBT with the effectiveness of g-CBT for treatment of depression. Our primary hypothesis is that g-RFCBT will be superior to g-CBT in reducing depressive symptoms measured on the score on the Hamilton Rating Scale for Depression (HRSD) at the end of treatment. Our secondary hypothesis is that g-RFCBT will be superior to g-CBT in reducing relapse at 6-month follow-up after the end of treatment. Relapse is defined as a score of 13 points or more on the HRSD.

### Eligibility criteria

Patients who are between 18 and 65 years of age and who meet Diagnostic and Statistical Manual of Mental Disorders, 4th Edition (DSM-IV) criteria for a current episode of unipolar major depression or recurrent or chronic depression in a structured M.I.N.I. 5.0 interview and with a score of 13 or more on the 17-item HRSD can be included [18]. Patients with psychotic symptoms, bipolar disorder, functional illiteracy, or alcohol and drug abuse will be excluded. Patients with depression and co-morbid anxiety or personality disorder are included in the study. Patients receiving anti-depressants will not be excluded.

### Recruitment

The study takes place in a community psychiatric outpatient service in Hillerød, Denmark, which treats 200–250 patients with diagnosed depression per year. Patients who are referred for treatment with a primary diagnosis of depression, recurrent or chronic, will be recruited to the study. We plan to recruit a total of 128 patients, 64 in each treatment arm.

The psychiatrist conducting the initial evaluation of the patients will invite the patients to talk to the researcher if the inclusion criteria are met. Written informed consent will be obtained from all patients prior to enrolment in the study. The researcher will validate the depression diagnosis and screen for DSM-IV Axis I co-morbidity by using the structured International Neuropsychiatric Interview M.I.N.I. version 5.0 [23] and for DSM-IV Axis II disorders by using the Standardized Assessment of Personality-Abbreviated Scale, an eight-item semi-structured interview screening for the presence of a personality disorder [24]. Patients meeting the inclusion criteria will be included in the study, and baseline assessment will be conducted within 2 weeks prior to commencement of the psychotherapeutic treatment. After the baseline assessment, the patients are randomly assigned to receive one of the two treatment options.

### Randomisation and concealment

Participants will be allocated in a 1:1 ratio to either g-CBT or g-RFCBT. The randomisation will be concealed from the investigator by use of an off-site computer-based randomisation. Patients are randomly assigned in blocks of variable sizes (6–10) according to a pre-study computer-generated (Microsoft Excel 2011; Microsoft Corporation, Redmond, WA, USA) randomisation list to receive one of the two treatment options.

All assessments will be conducted equally in both treatment conditions by researchers blind to randomisation. To successfully maintain the blinding throughout the trial, the researchers conducting the patient assessments will be instructed to remind the patients of the confidential nature of their treatment.

### Interventions

RFCBT is a manualised CBT treatment [18] with 12 individual sessions scheduled weekly or fortnightly. In this trial, the treatment will be adapted to a group format consisting of one individual session of 1 h followed by 11 group sessions of 3 h (with two breaks of 15 min) scheduled weekly or fortnightly. RFCBT is theoretically informed by the distinction between constructive and unconstructive forms of repetitive thinking [21]. The main purpose of the treatment is to coach individuals to shift from unconstructive rumination to constructive thinking and problem solving through the use of functional analysis, experiential/imagery exercises, and behavioural experiments. For example, individuals will practice shifting from a general, evaluative, and abstract way of thinking to a more specific, descriptive, and concrete style of thinking. The g-RFCBT consists of an individual session focusing on development of an idiosyncratic RFCBT model of depression and finding individual treatment goals followed by 11 group sessions. Session 1 consists of psychoeducation about the connection between mood and behaviour and an introduction of rumination as a maladaptive behaviour [22]. Sessions 2 and 3 consist of training in the use of functional analysis to identify maladaptive behaviours [25]. Session 4 consists of training in rational and stepwise problem solving [26]. Session 5 consists of training in shifting from a maladaptive abstract thinking style to a constructive and more specific thinking style [27]. Session 6 consists of training in being present and absorbed in activities [28]. Sessions 7 and 6 consist of training in building compassionate images and in the use of compassion in everyday life [29]. Session 9 consists of training how to evaluate personal progress without getting stuck in unconstructive thinking [28]. Session 10 consists of working on how to engage in a valuable life and avoid getting stuck in rumination. Session 11 consists of building resilience and psychoeducation on prevention of relapse [14].

The control group intervention is CBT based on Aaron Beck’s CBT manual for depression [27] but adapted to group format consisting of one individual session of 1 h followed by 11 group sessions of 3 h scheduled weekly or fortnightly, matched in duration, structure, and therapist contact with g-RFCBT.

In addition, all participants will be offered clinical management and treatment with anti-depressant medication if needed, as evaluated by a highly trained and experienced psychiatrist at the outpatient service. There are no restrictions in use of anti-depressant medication in this trial, but use of anti-depressive medication and changes in type of medication or dosage will be registered.

#### Intervention fidelity

Therapists conducting the g-RFCBT and the g-CBT therapies are trained cognitive behavioural therapists with at least 7 years of experience with CBT. The therapists conducting g-RFCBT groups are trained and supervised 1 h per month by the developer of RFCBT (ERW). The therapists conducting the g-CBT groups are supervised 1 h per month by a highly trained and experienced CBT supervisor (NKR). Supervision uses videotaped sessions discussed on Skype as well as face-to-face feedback on recorded sessions.

Adherence to the clinical manuals as well as competency in conducting the therapy will be assessed by using videotapes from therapy sessions. Experts in both treatments will rate a random 10 % sample of videotapes, stratified by therapy session and groups. Adherence to the manuals will be rated by using a checklist to assess the presence of key therapy components and the absence of prohibited components for both treatments. Competence will be rated by using the Cognitive Therapy Rating Scale (CTRS) for g-CBT [30] and an adjusted version of the CTRS for g-RFCBT. For both arms, kappa statistics on inter-rater reliability will be reported.

### Outcomes

#### Primary outcome

Primary outcome is the post-treatment assessment with the HRSD [31]. The HRSD is a standardised clinical interview developed to assess severity of depressive symptoms. The researcher conducting the interviews is trained in the procedure and blinded with regard to the treatment group.

#### Secondary outcomes

Secondary outcomes will be conducted post-treatment by using the following measures. The Hamilton Depression Rating Scale (HAM-D6) is a six-item self-report rating scale (corresponding to the 17-item observer-rated HRSD), which measures the core symptoms of the depression construct on a unipolar scale. The HAM-D6 questionnaire has been shown to be a reliable and valid measure of the severity of depressive symptoms in a clinical sample [32]. The Behavioural Activation for Depression Scale (BADS) is a 25-item questionnaire measuring changes in avoidance and activation over the course of behavioural activation and has been shown to be reliable and valid in a community sample with elevated depressive symptoms [33]. The Penn State Worry Questionnaire (PSWQ) is a 16-item questionnaire assessing the general trait of worry and has been shown to be reliable and valid in a clinical sample [34]. The Rumination Response Scale (RRS) is a 22-item self-report measure that assesses ruminative response to depressive symptoms. Participants are asked to indicate the frequency of rumination with the total score indicating the severity of rumination used as a strategy in response to depressive symptoms [35]. The Generalised Anxiety Scale (GAD-7) is a seven-item questionnaire assessing the severity of symptoms of generalised anxiety disorder. GAD-7 has been shown to be valid in a community sample [36]. The World Health Organization Well Being Index (WHO-5) is a five-item questionnaire assessing the level of well-being [37]. The Trail Making Task A and B is a neuropsychological test of visual attention and task switching. It consists of two parts in which the test respondent is asked to connect 25 dots as fast as possible while still maintaining accuracy [38]. The Dot Probe Task is a non-verbal computerized task assessing the level of visual attentional bias from emotional pictures [39]. Furthermore, assessment with Dot Probe Task, HAM-D6, RSS, PSWQ, and BADS will take place after the fourth and the eighth sessions. A 6-month post-treatment follow-up will be conducted to measure the level of depressive symptoms with HRSD, and depressive relapse will be assessed by a diagnostic interview based on DSM-IV criteria for depression.

### Adverse events

All adverse events will be monitored by the psychotherapists and the psychiatrists at the outpatient service and reported in the patient case report. If a patient is at risk of an adverse event, a psychotherapist, psychiatrist, or research staff will take immediate action to prevent any adverse event from happening. Use of anti-depressive medication will be administered and monitored by experienced psychiatrists at the outpatient service. There are no study-specific restrictions on change of dosage of or types of anti-depressant medications. Medications, adverse effects due to medication, and changes in medical treatment will be reported in the patient case record by the psychiatrists. We will compare the levels of usage of anti-depressant medication and the reported adverse effects across the arms by conducting a screening of the participants’ case reports when all participants have finished treatment.

## Statistical considerations

### Sample size estimation

Assuming similar mean changes in HRSD scores from pre- to post-intervention as found by Watkins and colleagues [18] for RFCBT (M = 7.81) and by Paykel and colleagues [11] for CBT (M = 3.52) and a conservative estimate of pooled standard deviation for change in HRSD of 6 (when standard deviation = 3.60 for change in HRSD in RFCBT), we estimate a between-treatment effect size of Cohen’s *d* = 0.7. To detect a difference in effect size of 0.7 between g-RFCBT and g-CBT at a two-tailed significance level of 5 %, each treatment arm requires 44 patients to obtain 90 % statistical power [40]. Assuming a dropout rate of 20 %, we will recruit 55 patients into each treatment arm. With an average size of the therapy group of *m* = 8 in both treatment arms and an intraclass correlation of about *ρ* = 0.05, a design effect of 1 + (*m* – 1)*ρ* = 1.35 follows, so that we plan to recruit eight groups in each treatment arm (128 patients in total).

### Data analysis

The primary outcome is the post-treatment score on the HRSD, which is treated as a continuous, normally distributed variable. The primary efficacy hypothesis will be tested by using a multilevel two-group comparison (g-RFCBT versus g-CBT), with group as a main effect, therapy group as a random intercept, and the HRSD baseline score as a continuous covariate [41]. The test will be performed at the 5 % two-tailed significance level [42].

The primary test for efficacy will be based on the intention-to-treat population with all randomised patients entering the analysis set. The multiple imputation method will be used for missing data when appropiate [42].

### Ethical considerations

We will conduct the trial in such a way as to protect the human rights and dignity of participants as reflected in the Helsinki Declaration [43]. The patients will be informed about the research project and purpose of the project prior to participation and asked for informed consent. No side effects due to the RFCBT have been reported. It is not possible to pay the patients for participating in the research project. We will follow good clinical practice in monitoring for suicide risk during all research and clinical encounters with trial participants. If any risk to participants due to expressed thoughts of suicide is encountered, we will report these directly to the psychiatrist responsible for treatment of the patient (with the participant’s expressed permission), and if an acute risk is present, we will arrange for an immediate assessment by the psychiatrist responsible for the treatment or follow the patient to the psychiatric emergency room for further assessment of need of hospitalization. The project has been approved by the Danish National Ethical Scientific Committee (registration number H-1-2013-049) and is registered at the Danish Data Protection Agency by the Mental Health Services of the Capital Region of Denmark.

## Discussion

Previous research supports rumination as a common residual symptom of depression that remains elevated after both partial and full remission. Elevated rumination is associated with diminished responsiveness to anti-depressant medication and cognitive therapy [44, 45], and rumination has been demonstrated as a crucial factor in vulnerability to depression, predicting the onset, severity, and duration of future depression [16]. The preliminary research on the effectiveness of RFCBT shows promising results compared with TAU [18]; furthermore, targeting rumination may improve relapse rates. In addition, no trial has directly compared g-RFCBT with g-CBT, and this randomised study may provide the first support for g-RFCBT as a potential alternative to g-CBT treatment. This may be relevant for patients who have not responded to CBT or for other reasons prefer a different, but still evidence-based, treatment for depression. The group format of delivering therapy is more cost-effective than individual therapy, which is widely used in CBT treatment for depression in outpatient services in Denmark. Service managers as well as the patients would most likely welcome a cost-effective treatment for depression with good outcome and reduced relapse rates.

This trial is conducted in a naturalistic setting on a heterogeneous sample of patients with recurrent depression, a single episode of depression, or chronic depression, and co-morbidity with anxiety and occasionally with personality disorder. The results are highly generalisable to the actual clinical reality in many outpatient services in Denmark. The treatment that is being tested in the present trial is robust as to heterogeneity and co-morbidity and even has potential as a transdiagnostic treatment for emotional disorders.

## Trial status

Recruitment commenced in October 2013 and is ongoing.

## Abbreviations

BADS: Behavioural Activation for Depression Scale
CBT: Cognitive Behavioural Therapy
CTRS: Cognitive Therapy Rating Scale
DSM-IV: Diagnostic and Statistical Manual of Mental Disorders, 4th Edition
GAD-7: Generalised Anxiety Scale
g-CBT: Group-based standard cognitive behavioural therapy
g-RFCBT: Group-based rumination-focused cognitive behaviour therapy
HAM-D6: 6-item Hamilton Depression Rating Scale
HRSD: Hamilton Rating Scale for Depression
PSWQ: The Penn State Worry Questionnaire
RFCBT: Rumination-focused Cognitive Behavioural Therapy
RRS: Rumination Response Scale
TAU: Treatment-as-usual
